# Optimization of Bokashi-Composting Process Using Effective Microorganisms-1 in Smart Composting Bin

**DOI:** 10.3390/s21082847

**Published:** 2021-04-18

**Authors:** Pei Sze Lew, Nik Nor Liyana Nik Ibrahim, Suryani Kamarudin, Norashikin M. Thamrin, Mohamad Farid Misnan

**Affiliations:** 1 Department of Chemical and Environmental Engineering, Universiti Putra Malaysia, Serdang 43400, Selangor, Malaysia; peisze.lew@gmail.com (P.S.L.); suryani@upm.edu.my (S.K.); 2 Agrosen Lab, Faculty of Electrical Engineering, Universiti Teknologi MARA, Shah Alam 40450, Selangor, Malaysia; norashikin@uitm.edu.my; 3 Faculty of Electrical Engineering, Kampus Pasir Gudang, Universiti Teknologi Mara Cawangan Johor, Shah Alam 40450, Selangor, Malaysia; mohamadfarid@uitm.edu.my

**Keywords:** bokashi composting, smart composting bin, WIFI, IoT

## Abstract

Malaysians generate 15,000 tons of food waste per day and dispose of it in the landfill, contributing to greenhouse gas emissions. As a solution for the stated problem, this research aims to produce an excellent quality bokashi compost from household organic waste using a smart composting bin. The bokashi composting method is conducted, whereby banana peels are composted with three types of bokashi brans prepared using 12, 22, and 32 mL of EM-1 mother cultured. During the 14 days composting process, the smart composting bin collected the temperature, air humidity, and moisture content produced by the bokashi-composting process. With the ATmega328 microcontroller, these data were uploaded and synchronized to Google Sheet via WIFI. After the bokashi-composting process was completed, three of each bokashi compost and a control sample were buried in separate black soil for three weeks to determine each compost’s effectiveness. NPK values and the C/N ratio were analyzed on the soil compost. From the research, 12 mL of EM-1 shows the most effective ratio to the bokashi composting, as it resulted in a faster decomposition rate and has an optimum C/N ratio. Bokashi composting can help to reduce household food wastes. An optimum amount of the EM-1 used during the bokashi-composting process will produce good quality soil without contributing to environmental issues.

## 1. Introduction

Municipal solid waste (MSW) is generated from households and public areas, such as residential areas, streets, or parks. Food wastes, home appliances, food packaging, and electronics components are examples of municipal solid waste. Approximately 90% of MSW in Malaysia is commonly disposed of in landfills and mostly by open dumping [1]. In 2015, the Solid Waste Corporation of Malaysia (SWCorp) stated that Malaysians generate 38,000 tons of waste per day; from this amount, around 15,000 tons is food waste [2]. Moreover, Johari et al. (2014) stated that food waste accounts for most solid waste in Malaysia, which undergoes complex decomposition [3]. Landfill gases generated from the decomposition process consist mainly of methane (CH4) and carbon dioxide (CO_2_), approximately 60 and 40%, respectively, contributing to greenhouse gas emissions [4].

Fruit wastes such as rotten fruits and fruit peels are categorized under food wastes. In 2017, banana became the most produced fruit in Malaysia with around 350,000 metric tons [5]. As the second-highest consumed fruit in Malaysia per capita, with 10.0 kg/year [6], banana becomes one of the major food waste sources. Statistics show that more than 72 percent of food waste can undergo decomposition without landfilling [7]. Household composting is one strategy to reduce food waste in landfills, as stated in [8]. According to Hanyong et al. (2020), household composting possesses lower impacts on global warming potential than landfills [9]. One of the household composting techniques is bokashi composting. Bokashi is the product from an anaerobic process using organic matter, effective microorganisms (EM), molasses, and water. Professor Teruo Higa developed bokashi composting using EM at the University of Ryukyus in Okinawa in 1982 [10]. There has been growing interest in food waste recycling using the bokashi composting method [11,12,13,14]. The studies reported that bokashi improves soil fertility, increases crop yield, and promotes plant growth. Besides that, bokashi as organic materials can increase the water-holding capacity of the soil [15]. The review by [16] summarized the advantages of applying the bokashi in agriculture as an organic fertilizer.

However, there are lesser food waste composting practices in the Malaysian household area. It is inconvenient to most working individuals who lack time to check on the composting process frequently. This scenario can be related to the residents in the urban area, as most of their time is compromised in working and driving in grave traffic conditions. Besides, the residents also have limited spaces to conduct the conventional composting method for food waste decomposition, as most of them are staying at apartments, condominiums, and double-story houses.

During the research, a smart bokashi composting bin with temperature, humidity, moisture, and water level sensor was built to produce good quality compost from household organic wastes. Besides that, the sensors’ conditions can be real-time synchronized with the use of the internet. With the on-site data synchronizing, the smart composting bin can inform users of the composting bin condition, allowing the user to access data such as the temperature, humidity, moisture, and water level in real time. The user can manage the bokashi-composting process by accessing the temperature, humidity, moisture, and water level data by utilizing the concept of the Internet of Things (IoT) technology to integrate the electronics sensors with each of the automated mechanisms via Internet WIFI connection. In this research, the banana peels are used as food wastes to undergo bokashi composting in the smart composting bin. Using different ratios of the EM-1 in the inoculant, the bokashi quality is compared and analyzed in nitrogen, phosphurs and potassium (NPK) values and C/N ratio.

## 2. Materials and Methods

**Figure 1:**
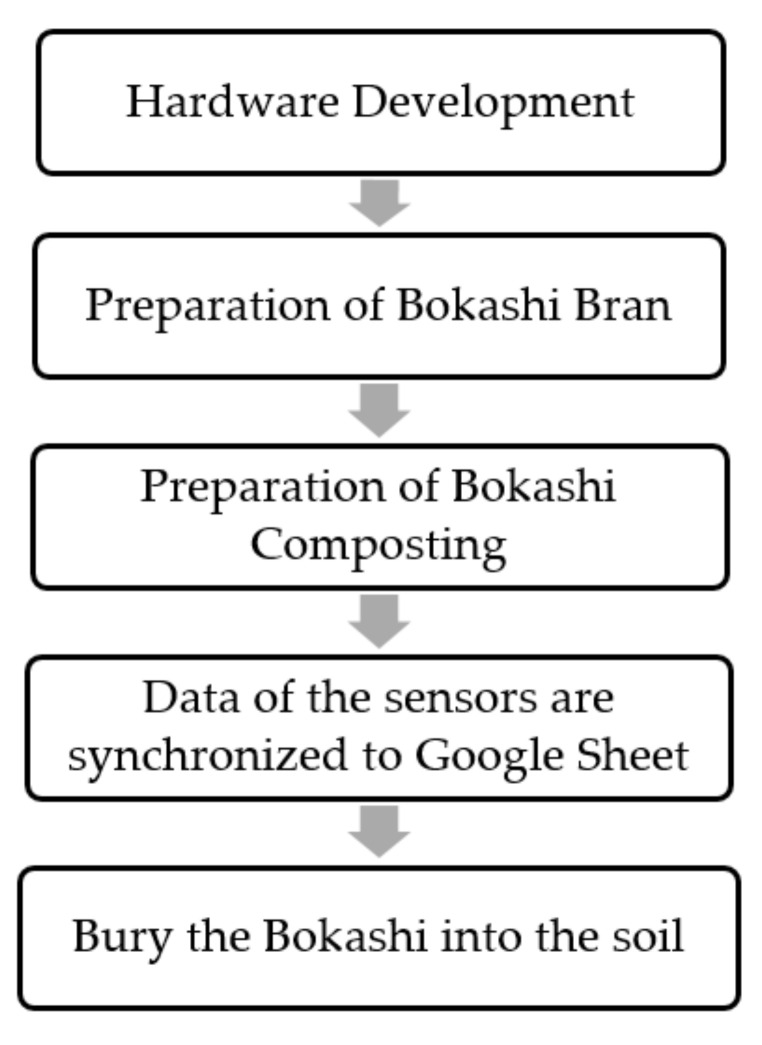
Overall process flow of this research.

The overall methodology is shown in Figure 1. Firstly, the smart bokashi bin was designed and fabricated using an ultrasonic sensor, a DHT11 humidity and temperature sensor, a water level sensor, a capacitive soil moisture sensor, an ATmega328 microcontroller, and an Arduino WIFI Shield ESP8266. Besides, the bokashi bran is required to be produced based on the formulation of wheat bran, effective microorganism, molasses, and water. After completing the bokashi bran preparation, the bokashi-making process can be started by adding the bokashi bran to the organic wastes. The bokashi-composting process was undergoing 14 days. The sensors’ parameters were uploaded and synchronized to Google sheet at every 1-h interval along the bokashi-composting process.

### 2.1. Hardware Development

The bokashi-composting bin (L 25 × W 25 × H 25 cm) made by polypropylene (PP) was purchased from an online platform [17]. A few sensors were added to the composting bin, including an ultrasonic sensor, capacitive soil moisture sensor, temperature and humidity sensor (DHT11), and water level sensor. The sensors were connected to the ATmega328 microcontroller, whereby this microcontroller is attached to the ESP8266 WIFI Shield. A few materials (Tiffany blue color) were printed using a 3D printer according to the design requirement. The specifications of the components are as shown in Table 1.

**Figure 2:**
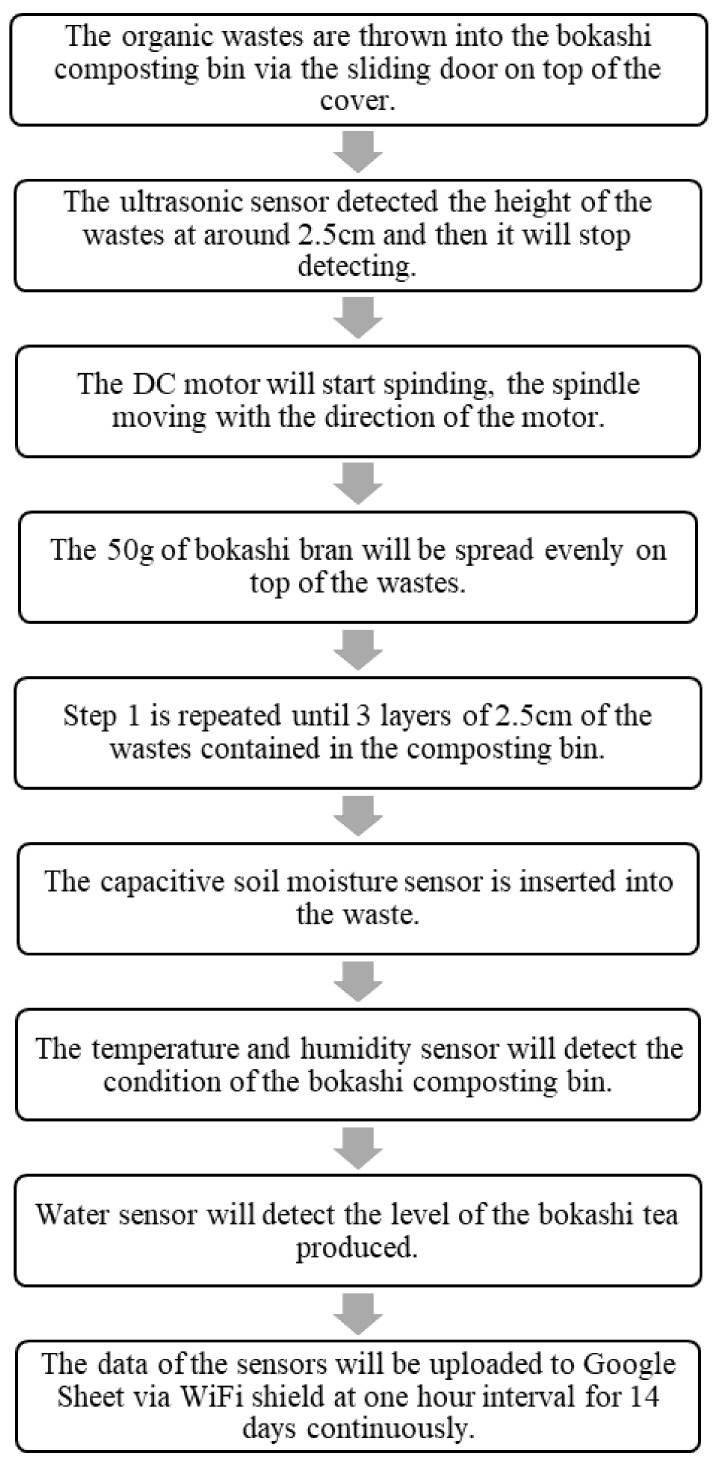
The operating procedures of the bokashi composting bin.

The organic waste chosen in this research was banana peels (*Pisang Nipah*). The banana peels were collected and cut into smaller pieces with dimension around 4 × 3 cm and disposed of into the smart composting bin until the ultrasonic sensor detects the 2.5 cm height of the banana peels layer. The number of banana peels per layer is not specified in this research, thus by estimating, a layer of 2.5 cm of banana peels will be equivalent to 400 g. The 50 g bokashi bran from the funnel is sprinkled through the spindle onto the banana peels until the spindle is stopped. The steps of banana peels being disposed of to the composting bin and the bokashi bran sprinkled to the composting bin with similar quantities were repeated until three layers were achieved. The detailed operating procedures of the smart composting bin are shown in Figure 2.

**Figure 3:**
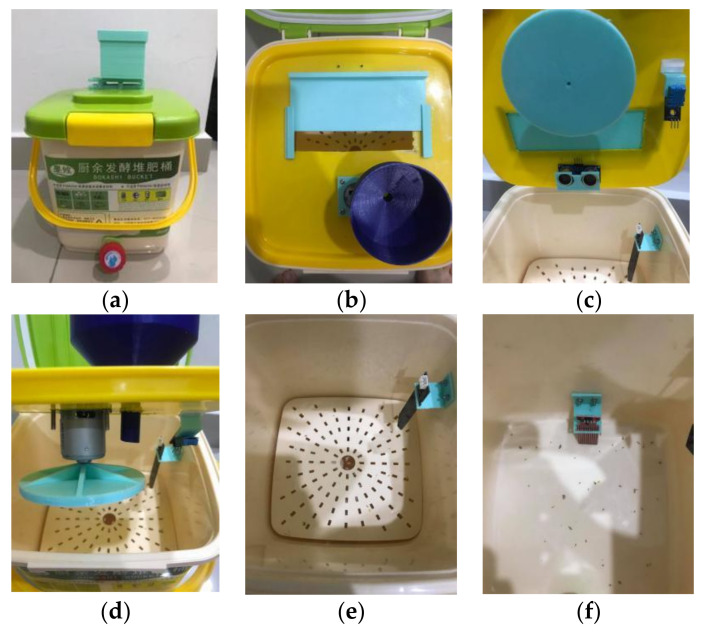
The components of the smart composting bin. (**a**) Shows the external view of the smart composting bin; (**b**) shows the additional cover on the composting bin; (**c**) shows the ultrasonic sensor and temperature and humidity sensor at the backside of the cover; (**d**) shows the DC motor and the spindle attached on the cover; (**e**) shows the soil moisture sensor attached on the wall of the bin; (**f**) shows the water level sensor attached to the bottom of the composting bin.

The control panel’s housing was put on top of the composting bin to protect the ATmega328 microcontroller and the ESP8266 WIFI Shield from contact with the moisture environment produced from bokashi, which is shown in Figure 3a. Besides that, after opening the composting bin’s cover, there was an additional cover on the bin. On the cover, a rectangular hole was made for wastes disposal, with a sliding door to ensure the hole was covered. Moreover, a funnel-shape-like holder helped to hold the bokashi bran, which would be sprinkled onto the organic waste. Figure 3b shows the additional cover on the bokashi composting bin. There were two sensors at the bottom of the cover: ultrasonic sensor and temperature sensor.

Figure 3c shows the ultrasonic sensor and temperature and humidity sensor. The ultrasonic sensor helps to detect the distance or the level of the organic wastes. Once the wastes reach around 3 cm in layer height, the ultrasonic sensor will stop and reset again after the bokashi bran was sprinkled onto the organic wastes. The new distance will be detected again. The temperature and humidity sensor helps to detect the current temperature and humidity of the bin. There was a DC motor attached to the cover of the bin. A round size spindle was attached to the DC motor, as in Figure 3d. When the bokashi bran from the funnel dropped to the spindle, the DC motor turned the spindle, and thus, the bokashi bran would be sprinkled evenly onto the organic wastes. The amount of the bokashi bran can be set according to the requirement. In this study, the amount of bokashi bran was set at 50 g, because 50 g is appropriate to cover a layer of the organic waste in this bin. Therefore, the amount of 50 g of bokashi bran is used to standardize the research project. After that, Figure 3e shows the moisture sensor attached to the wall of the composting bin. The moisture sensor will be inserted into the organic wastes once the wastes occupied 2/3 of the bin. Lastly, Figure 3f shows the water level sensor attached at the bokashi bin’s bottom. The water level sensor helps to measure the bokashi tea produced from the bokashi-composting process.

The sensors are connected to the ATmega328 microcontroller, and the sensors’ parameters are uploaded to Google Sheet at 1 h interval using Arduino WIFI Shield. The Arduino WIFI Shield model ESP8266 was attached to the ATmega328 microcontroller, as shown in user instructions. The function of Arduino WIFI Shield ESP8266 allows the ATmega328 microcontroller to connect to any WIFI connection. Hence, the data collected from the sensors can be uploaded to the Internet via WIFI protocol. The pins of the Real-Time Clock were connected to pin 5V, Ground, SDA, and SCL. SDA pin and SCL pin were dedicated to the ATmega328 microcontroller board. Besides that, the signal of the temperature and humidity sensor was connected to pin A0.

**Figure 4:**
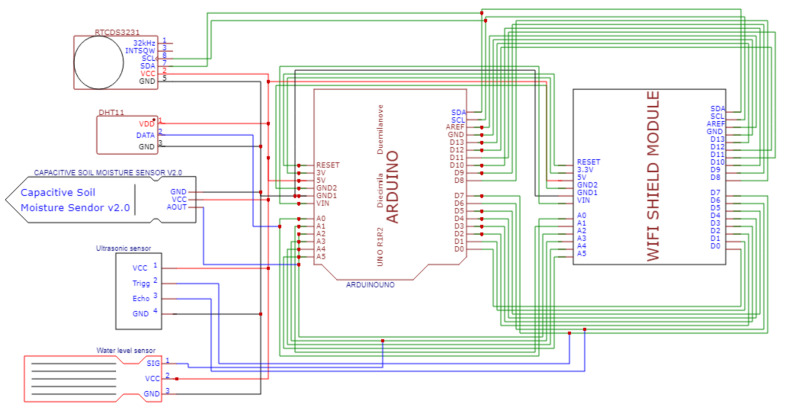
The schematic diagram of the circuit connection of the sensors.

Moreover, the capacitive soil moisture sensor’s signal was connected to pin A2, while the signal of the water sensor was connected to pin A3. Other than that, the pins of trigger and echo in ultrasonic sensors were connected to D7 and D6, respectively. Schematic diagram of the circuit, is as shown in Figure 4.

In this research, Google Spreadsheet acts as a database to record and show the data collected from the sensors. Application Programming Interface (API) is an interface to allow two applications to talk to each other. The data from sensors such as the DHT11 humidity and temperature sensor, water level sensor, and capacitive soil moisture sensor will be transmitted and displayed at Google Spreadsheet via the API. The Arduino WIFI Shield’s function is to allow the ATmega328 microcontroller board to connect to the internet using WIFI. Wire wrap headers of the Arduino WIFI shield allow it to connect to the ATmega328 microcontroller by stacking on top of the ATmega328 microcontroller.

### 2.2. Preparation of Bokashi Bran

**Figure 5:**
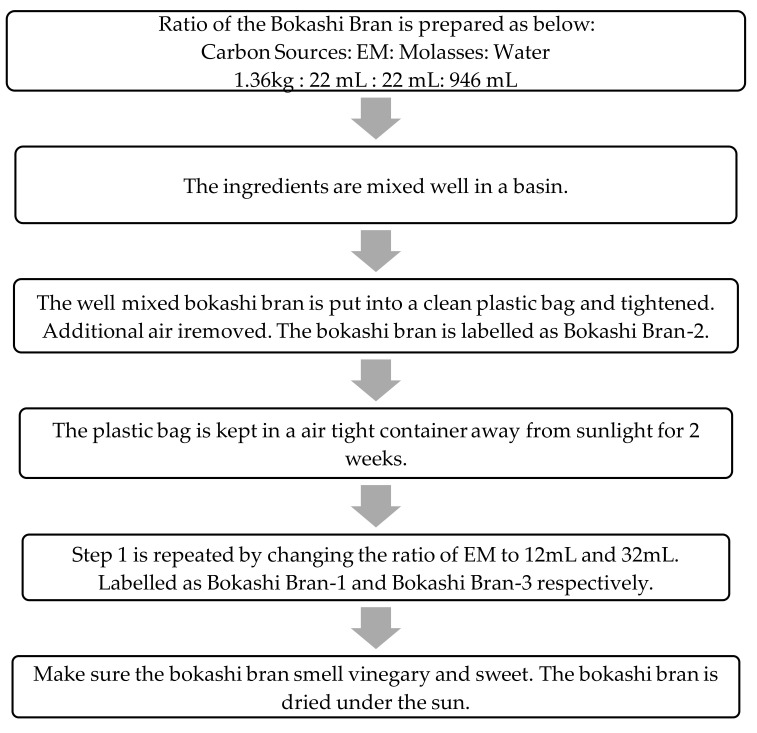
The process flow of the preparation of bokashi brans.

Bokashi bran preparation is one of the essential factors contributing to the success of the bokashi-composting process. The detailed process flow of the bokashi bran preparation is shown in Figure 5.

Three types of bokashi bran were produced using a similar medium but different formulation, as shown in Table 2. The amount of the EM-1 and molasses was fixed at 1 to 1 ratio in order to ensure no effects of molasses on the bokashi formulation. The usage of molasses only was to enhance the growth of microorganism during the bokashi-composting process. With this formulation, the relationship between EM-1 volumes with the carbon source (wheat bran) will be determined and analyzed.

### 2.3. The Procedure of Soil Buried with Bokashi

The soil composting is carried on after the bokashi composting. Soil composting with bokashi will help to identify each type of bokashi nutrient value, such as nitrogen, phosphorus, and potassium. The bokashi, also known as pre-compost, was buried into the black soil for further composting process.

After the completion of the bokashi-composting process, the bokashi was taken out from the composting bin. A 2.4 kg amount of the black soil was prepared and poured into a 15 L plastic bin. A hole was dug from the soil, and the bokashi was disposed into the soil and buried for three weeks. The soil was turned once a week to check the condition of the bokashi. The samples were labeled as Soil Sample 1, Soil Sample 2, and Soil Sample 3, according to Bokashi-Bran-1, Bokashi-Bran-2, and Bokashi-Bran-3, respectively. Besides that, sample 4 only consists of plain banana peels buried into the soil without undergoing the bokashi-composting process. It acts as a control to compare the results with the soil samples from the bokashi-composting process.

### 2.4. Analytical Method for Soil Analysis

In this study, the NPK value and the C/N ratio of the soil will be analyzed. The total nitrogen of the soil was analyzed using the Kjeldahl method. The soil’s carbon was analyzed using the APHA 5310B method, which can detect the total organic carbon in the soil. Besides, the potassium and phosphorus were measured by using USEPA 3050B. This method is internationally recognized to analyze solid sample elements, such as soil, sediment, and sludge [24]. The analytical test method was conducted by a chemist under ChemVi Laboratory Sdn Bhd, located at Shah Alam, Selangor.

## 3. Results

### 3.1. Intra-Bokashi Composting Period

The data of the bokashi moisture content and the bokashi bin’s temperature and air humidity were collected in the enclosed bokashi bin during the bokashi-composting process. The data were collected for 14 days at every one-hour interval.

**Figure 6:**
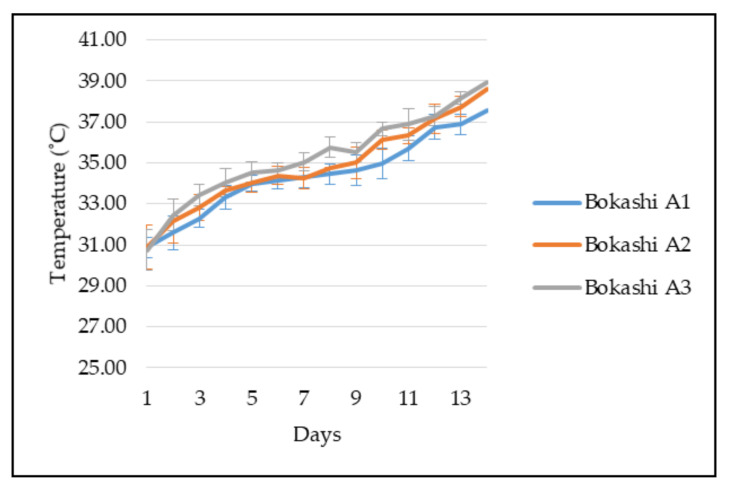
The observation of the temperature of different types of bokashi from day 1 to day 14.

According to Figure 6, the Bokashi A3 in the composting bin was maintained at the highest temperature from the second day of the bokashi composting compared to the other two bokashi. The highest temperature of the Bokashi A3 was 39 °C on the 14th day. Next, the Bokashi A1 recorded the lowest temperature among the bokashi during these 14 days of the bokashi-composting process. The highest temperature of the Bokashi A1 obtained was 38 °C. The temperature of the three EM bokashi increased gradually from Day 1 until Day 14. However, there were no obvious differences in terms of temperature variation among the three bokashi observed. The increased temperature indicated that the bokashi-composting process is in progress.

**Figure 7:**
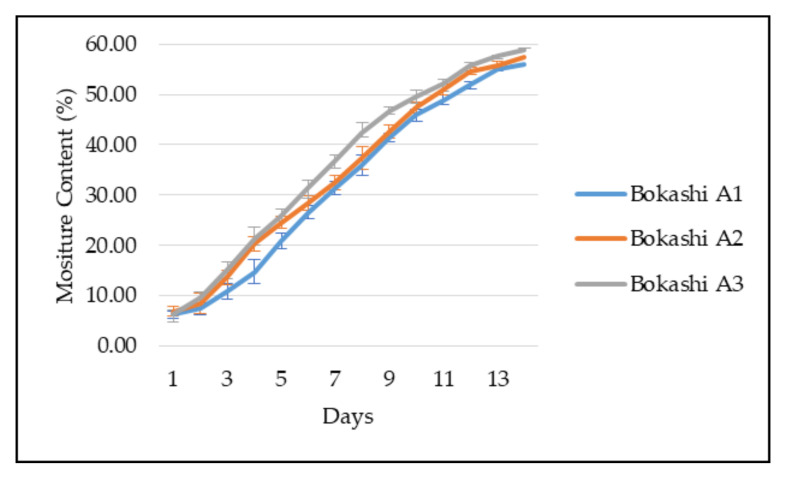
The observation of the moisture content of different types of bokashi from day 1 to 14.

Moreover, Figure 7 shows the moisture content of the bokashi during the bokashi-composting process. In this figure, the moisture content of Bokashi A3 started at 6.38% on the first day. The increment of moisture content of Bokashi A3 started to slow down on at 10th day and finally achieved 58.75% on the 14th day. Next, Bokashi A2 obtained 6.88% on the first day and 57.33% on the final day. Bokashi A1 measured 6.25% on the first day and 55.92% on the 14th day; thus, it shows the slowest moisture content increment rate.

**Figure 8:**
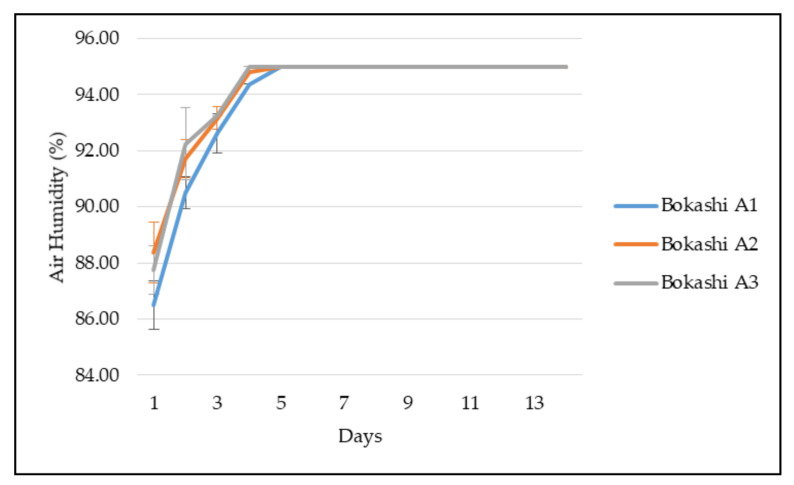
The air humidity observation in the bokashi composting bin of different bokashi types from day 1 to day 14.

Besides, Figure 8 shows the air humidity condition in the composting bin during the bokashi-composting process. Bokashi A1 shows the lowest air humidity during the first day, which was 86.50%. The humidity increased until it reached a constant value of 95% on the 5th day. On the other hand, the Bokashi A2 measured 88.38% on the first day, which was the highest value among the bokashi compost. The Bokashi A1 increased gradually and reached a constant value of 95% on day 5. Unlike Bokashi A1 and Bokashi A2, Bokashi A3 obtained 87.75% of air humidity on the first day and increased on the subsequent day, until it reached a constant value of 95% since day 4.

### 3.2. Soil Samples after Bokashi Composting

A total of four samples were being tested for C/N ratio, nitrogen, phosphorus, and potassium values. The samples were the soil buried with the Bokashi A1 (Soil Sample 1), soil buried with the Bokashi A2 (Soil Sample 2), soil buried with the Bokashi A3 (Soil Sample 3), and Soil Sample 4 as a control sample. The control sample is a sample that buried the organic wastes without additional the bokashi bran into the soil.

Table 3 shows the results of the C/N ratio of each of the samples. According to the table, Sample 2 obtained the highest ratio, which is 27.32. Meanwhile, Sample 1, Sample 3, and Sample 4 (Control) have the C/N ratio of 25.39, 25.36, and 20.22, respectively.

On the other hand, the banana phosphorus is recorded in the unit of parts per million (ppm) in the report. After conversion of the unit from ppm to percentage, Sample 1, Sample 2, Sample 3, and Sample 4 consisted 0.0758, 0.096, 0.1131, and 0.0645%, respectively. Table 4 shows the phosphorus content of the samples. Moreover, the same as the phosphorus results, the potassium unit was converted from ppm to percentage and shown in Table 5. Among the results, Sample 1 has the highest value, which is 0.5883%, followed by Sample 4, Sample 2, and Sample 3, with the values of 0.4089, 0.3894, and 0.3673%, respectively.

## 4. Discussion

### 4.1. Intra-Bokashi Composting Period

On average, the temperature of the bokashi increased by 1 °C in a day. The maximum temperature of the bokashi was recorded at 39 °C. The EM culture in the bokashi bran includes yeast, lactic acid bacteria, and phototrophic bacteria. The optimum temperature for the yeast to grow is at the optimum temperature (32.3 °C) and a maximum growth temperature (45.4 °C) [25]. The optimum growth temperature for lactic acid bacteria is between 37 and 40 °C [26]. While the optimum growth temperature of phototrophic bacteria is around 37 °C, and the maximum growth temperature is around 45 °C [27]. The temperature of bokashi in the graph showed that the EM grows in the optimum condition and does not exceed the microorganisms’ maximum growth temperature, which ensures that the microorganisms were in healthy and appropriate conditions. Bokashi A3 obtained the highest temperature, and the lowest temperature was obtained by Bokashi A1, due to the concentration of the EM-1 in the formulation. Bokashi A3 has the most concentrated effective microorganisms in the bokashi-composting process, while Bokashi A1 contains the least effective microorganisms. As a result, the higher concentration of effective microorganisms will cause more microbes’ respiration and increase the temperature.

The moisture content of the three bokashi types increased gradually during the bokashi-composting process. The bokashi’s initial moisture content was around 6 to 7%, which comprises of the banana peels’ moisture content during that particular time. After ten days, Bokashi A3 achieved 50% moisture content, while Bokashi A2 and A1 recorded 50% moisture content on the eleventh and twelfth day, respectively. The moisture content of the bokashi does not exceed 60% during the bokashi-composting process. According to research, banana peels (Musa spp.) have a mean value of 50.5% of moisture content in a wet sample [28]. Research shows the compost’s optimum moisture content should range from 50 to 60%, while the compost is in the active phase [29]. Over time, the moisture content of the bokashi increased as the temperature increased. The moisture released from the banana peels was condensed in the closed vessel of the composting bin. Thus, the internal walls of the composting bin were wet as water condensed.

The initial air humidity in the composting bin’s closed system was high, measured at 84 to 89% on the first day. Malaysia is a tropical country with warm and high humid weather conditions. The air humidity of Malaysia was recorded at 74 to 86% annually [30]. The humidity of the bokashi bin’s closed system achieved a maximum of 95% air humidity on the fourth day and the fifth day as the bokashi’s moisture increased. It was believed that all organic waste performed degradation during this time, and the air in a closed bin was saturated. The air humidity values were stagnant until day 14.

There were supposed additional data expected to be collected during the bokashi-composting process, the bokashi tea level. This bokashi tea was believed to be produced during the bokashi-composting process. However, it was out of expectation that no bokashi tea was produced during the bokashi-composting process, as three bins mentioned that no bokashi tea would be produced if too much of the bokashi bran is added into the organic waste [31]. However, it will not affect the results of the bokashi composting.

### 4.2. Soil Samples after Bokashi Composted

**Figure 9:**
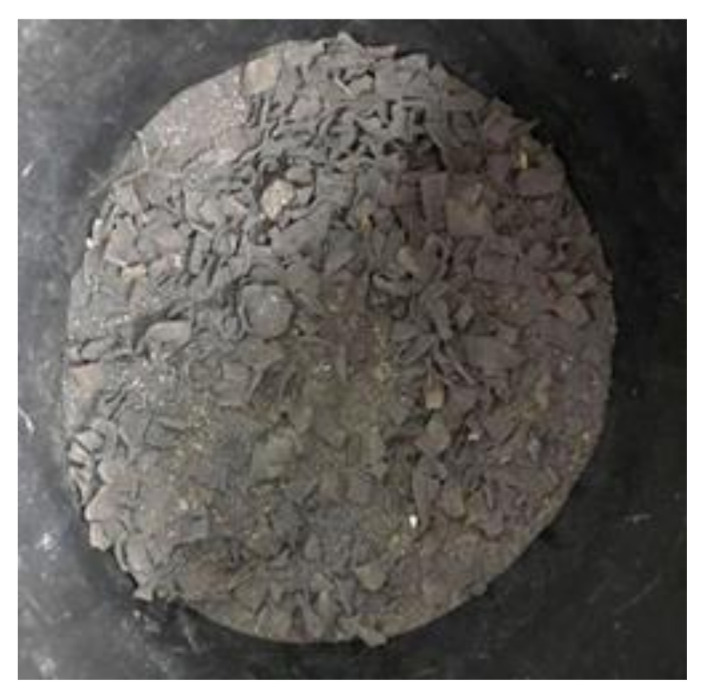
Soil sample after 21 days of soil composting.

Microorganisms consumed carbon and nitrogen as sources of nutrients. Microorganisms utilized carbon as the energy source to grow and nitrogen to build up the cell structures [32]. As the composting process period increases, the amount of carbon and nitrogen will decrease, since the microbes grow continuously. The carbon value and nitrogen value obtained from the result were used to calculate the C/N ratio for all the samples. Sample 4 contained the lowest C/N ratio at 20.22. Without having the additional microbes added during the soil composting, Sample 4 could be associated with a low carbon loss rate and nitrogen loss, since less carbon and nitrogen were consumed for microbe growth. Figure 9 shows the condition of the soil after 21 days of soil composting. From the figure, the banana peels were dried and not fully decomposed into the soil. Thus, it was proven that there was still a high value in the carbon and nitrogen in Sample 4.

In contrast, Sample 1, Sample 2, and Sample 3 have the C/N ratio of 25.39, 27.32, and 25.36, respectively. The ideal C/N ratio falls in the range between 25–35, and it is essential to ensure an efficient compost mix [32,33]. When the C/N ratio is above the optimal range, the composting process becomes slow. In this scenario, carbon content is relatively higher than nitrogen content. Due to limited nitrogen content, microorganisms require a longer time to utilize the excess carbon. It requires several life cycles of microorganisms to lessen the C/N ratio to a more appropriate level [32].

**Figure 10:**
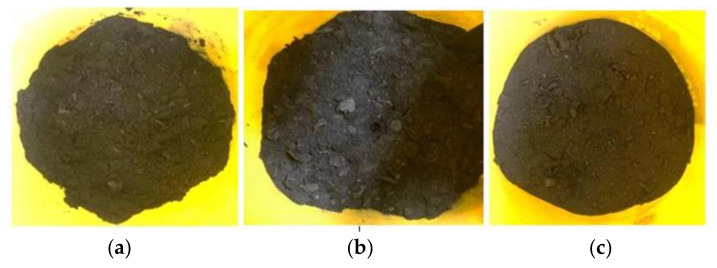
Soil samples after 21 days of soil composting. (**a**) Soil Sample 1 (Bokashi A1); (**b**) Soil Sample 2 (Bokashi A2); (**c**) Soil Sample 3 (Bokashi A3).

On the contrary, if the C/N ratio is lower than the optimal range, the limiting nutrients will be the carbon, limiting the energy source provided to the microorganism for survival. Hence, it can be noticed that Sample 1, Sample 2, and Sample 3, which contained bokashi bran with EM-1, provide the favorable C/N ratio for microbe growth. Besides that, a study shows that EM’s application into the soil will increase the soil microbial biomass C and N and soil respiration [34]. Figure 10 shows the condition of Sample 1, Sample 2 and Sample 3 after 21 days of soil composting.

Figure 10a shows the banana peels in Soil Sample 1 were fully decomposed, and the soil was contaminated with water. Next, Figure 10b shows the banana peels in Soil Sample 2 were still undergoing the decomposition process. However, the soil was moist by observation. Moreover, Soil Sample 3 in Figure 10c shows that the banana peels were also decomposed, but it was observed that the soil was drier than Soil Sample 1. Thus, Figure 10a–c could be used to explain the C/N ratio of the soil samples obtained.

The phosphorus value obtained from the samples was closed to each other, with the descending order of Sample 3 > Sample 2 > Sample 1 > Sample 4. Averagely, the soil contains 0.05% of the phosphorus [35]. The phosphorus concentration in the soil samples increases as the organic wastes lose their nutrients to the soil. The small variation in the percentage of phosphorus might be due to the inconsistent weight of banana peel waste used during the bokashi-composting process. Moreover, Sample 1 contains the highest potassium value, which is 0.51%, followed by Sample 4 (0.41%), Sample 2 (0.39%), and Sample 3 (0.37%). Potassium and phosphorus increases in the soil samples could be due to the higher loss rate of carbon during the decomposition of organic wastes or mineralization processes into methane or carbon dioxide [36]. Compared to the phosphorus value, the soil samples’ potassium value was high due to banana peels’ high inherent content [37].

## 5. Conclusions

After studying the results obtained from the research, it was found that among the bokashi bran samples, Bokashi Bran-1, with 12 mL of the molasses, 1.36 kg of the wheat bran, and 946mL of water tends to obtain more favorable results. Most of the articles and books had mentioned that the bokashi bran should be mixed in the ratio of 22 mL of EM-1, 22 mL of molasses, and 946 mL of water in 1.36 kg of the bran. However, the suggested method is commonly applied to the western country, which has a relatively lower annual average temperature and humidity than Malaysia. Thus, this study has found that with the lower amount of EM-1 mother cultured, the organic wastes can be decomposed faster than using a higher amount of EM-1 mother cultured.

Besides that, the usage of the smart composting bin for analyzing the condition of the bokashi-composting process would help to reduce the chances of failing the bokashi-composting process. By utilizing the sensors attached to the bokashi composting bin, the bokashi quality will be improved. Based on results and discussion, organic waste is suggested to undergo bokashi composting for around 10 to 11 days compared to the duration used in this research, 14 days. The reason is that the bokashi moisture content started to increase after day 11, and black mold will form if the bokashi condition is extremely moist. Moreover, the temperature sensor will help ensure the process is progressing continuously, as it has shown that the temperature increases over time. Lastly, methane gas was produced in an anaerobic process; further research is recommended to determine the amount of methane production during the bokashi composting.

Recently, waste management is a concerning issue in Malaysia, especially household wastes, which consist of mainly organic wastes, contributing to the greenhouse effects in the long term. Household bokashi composting is an effective way to reduce the quantity of organic waste sent to landfills. Besides, using the optimum quantity of the EM-1 during the bokashi-composting process, good-quality soil can be produced and applied to the planting area, thus improving the soil’s condition without contributing adverse effects to the environment.

## Figures and Tables

**Table 1:** Specifications of the components.

Components	Specifications
ATmega328 microcontroller [18]	Operating voltage: 2.7 V to 5.5 V; Operating Current: 1.5 mA; Speed grade: 0 to 8 MHz at 2.7 to 5.5 V; I/O and packages: 23 programmable I/O lines : 32-lead TQFP, and 32-pad QFN/MLF.
ESP8266 WIFI Shield [19]	802.11 b/g/n wireless standards; Pin-compatible with Arduino UNO, Mega; Arduino Pinout 2/3/4/5/6/7/8/9/10/11/12/13; ESP8266 GPIO Pinout 0/2/4/5/9/10/12/13/14/15/16/ADC/EN/* UART TX/UART RX; Dual-Ports DIP switches: switching Arduino and ESP8266; WIFI operation current: continuous transmission operation: ≈70 mA (200 mA MAX), idle mode: <200 uA; Serial WIFI transmission rate: 110–460800 bps; Temperature: −40 °C~+125 °C.
Ultrasonic Sensor [20]	Operating Voltage: +5 VDC; Quiescent Current: <2 mA; Operating Current: 15 mA; Effectual Angle: <15°; Ranging Distance: 2~400 cm; Resolution: 0.3 cm; Measuring Angle: 30°; Trigger Input Pulse width: 10 uS.
DHT11 Humidity and Temperature Sensor [21]	Operating Voltage: 3.3~5.5 VDC; Operating Current: 0.5~2.5 mA; Measurement Range: 20–90%RH & 0–50 °C; Response Time: 6~15 s.
Capacitive Soil Moisture Sensor [22]	Operating Voltage: 3.3~5.5 VDC; Operating Current: 5 mA; Interface: PH2.0-3P.
Water Level Sensor [23]	Operating Voltage: 5 VDC; Operating current: <20 mA; Detection Area: 40 × 16 mm; Operating Temperature: 10~30 °C; Operating Humidity: 10~90%.

**Table 2:** The composition and moisture content of the bokashi bran.

Bokashi Bran	Composition				Moisture Content
	EM-1	Molasses	Wheat Bran	Water	
Bokashi Bran-1	12 mL	12 mL	1.36 kg	946 mL	42.1%
Bokashi Bran-2	22 mL	22 mL	1.36 kg	946 mL	42.4%
Bokashi Bran-3	32 mL	32 mL	1.36 kg	946 mL	42.3%

**Table 3:** C/N ratio of each soil sample after 21 days decomposition of bokashi.

Soil Sample	C/N Ratio of Each Sample
Sample 1	25.39
Sample 2	27.39
Sample 3	25.36
Sample 4	20.22

**Table 4:** The phosphorus percentage of the soil samples after 21 days decomposition of bokashi.

Soil Sample	Phosphorus Percentage of Each Sample
Sample 1	0.08%
Sample 2	0.10%
Sample 3	0.11%
Sample 4	0.06%

**Table 5:** The potassium percentage of the soil samples after 21 days decomposition of bokashi.

Soil Sample	Potassium Percentage of Each Sample
Sample 1	0.58%
Sample 2	0.39%
Sample 3	0.37%
Sample 4	0.41%

## Data Availability

Not applicable.
